# Side Effects of COVID-19 Pfizer-BioNTech mRNA Vaccine in Children Aged 12–18 Years in Saudi Arabia

**DOI:** 10.3390/vaccines9111297

**Published:** 2021-11-09

**Authors:** Edrous Alamer, Abdulaziz Alhazmi, Naaif A. Qasir, Rahaf Alamer, Halima Areeshi, Gassem Gohal, Marwa Qadri, Anwar M. Hashem, Abdullah Algaissi

**Affiliations:** 1 Department of Medical Laboratory Sciences, Faculty of Applied Medical Sciences, Jazan University, Jazan 45142, Saudi Arabia; ealamer@jazanu.edu.sa (E.A.); naaifq1@hotmail.com (N.A.Q.); 2 Emerging and Epidemic Infectious Diseases Research Unit, Medical Research Center, Jazan University, Jazan 45142, Saudi Arabia; abalhazmi@jazanu.edu.sa (A.A.); rahafalamer99@gmail.com (R.A.); halimaareeshi@hotmail.com (H.A.); mqadri@jazanu.edu.sa (M.Q.); 3 Microbiology and Parasitology Department, College of Medicine, Jazan University, Jazan 45142, Saudi Arabia; 4 College of Medicine, Jazan University, Jazan 45142, Saudi Arabia; 5 Department of Pediatrics, College of Medicine, Jazan University, Jazan 45142, Saudi Arabia; dr.gassem@gmail.com; 6 Department of Pharmacology and Toxicology, College of Pharmacy, Jazan University, Jazan 45142, Saudi Arabia; 7 Department of Medical Microbiology and Parasitology, Faculty of Medicine, King Abdulaziz University, Jeddah 21589, Saudi Arabia; amhashem@kau.edu.sa; 8 Vaccines and Immunotherapy Unit, King Fahd Medical Research Center, King Abdulaziz University, Jeddah 21589, Saudi Arabia

**Keywords:** SARS-CoV-2, COVID-19 pandemic, COVID-19, SARS-CoV-2, vaccine, Pfizer-BioNTech, side effects, children

## Abstract

**Background**: Massive vaccination campaigns have been undertaken globally to combat the spread of the Coronavirus Disease 2019 (COVID-19). While most COVID-19 vaccines have shown excellent efficacy and safety profiles in clinical studies, real-world monitoring of vaccine safety is still important. In this study, we aimed to investigate the early side effects of Pfizer-BioNTech (BNT162b2) mRNA vaccine in children between 12–18 years old in Saudi Arabia. **Method**: To investigate the side effects in children in this age range following the administration of either one or two doses of Pfizer-BioNTech (BNT162b2) mRNA vaccine, we conducted a retrospective, cross-sectional study using a self-administered online survey. General and demographic data were collected, and vaccine-associated side effects following vaccination were evaluated. **Results**: The study recruited a total of 965 eligible participants. Overall, 571 (60%) of the study participants reported at least one side effect following Pfizer-BioNTech (BNT162b2) mRNA vaccination. The most frequently reported side effects were pain or redness at the site of injection (90%), fatigue (67%), fever (59%), headache (55%), nausea or vomiting (21%), and chest pain and shortness of breath (20%). Joint or bone pain were reported less frequently among our participants (2%). Our data showed that more female participants reported side effects compared to male participants, with 52% and 48%, respectively. Side effects were more common after the second dose compared to the first dose in our study cohort. **Conclusions**: While 60% of the children (12–18 years old) who received Pfizer-BioNTech (BNT162b2) mRNA vaccine reported side effects, our data showed that these side effects were not different from those that were reported in the clinical trials which lasted only for a few days. Side effects were more common after the second dose. Larger epidemiological and molecular studies are needed to evaluate the safety and the effectiveness of COVID-19 vaccine in protection of children against SARS-CoV-2 reinfections.

## 1. Introduction

Coronavirus Disease 2019 (COVID-19) is an ongoing global pandemic that is caused by the novel severe acute respiratory syndrome coronavirus 2 (SARS- CoV-2) [1]. On 12th of March 2020, COVID-19 was declared as a global pandemic by the World Health Organization. Since then, more than 215 million confirmed cases and ~ 4.5 million deaths have been reported from all around the world [2]. SARS-CoV-2 infections can cause a wide variety of clinical manifestations that range from asymptomatic or mild to severe lung and multiorgan infections that can lead to death [3]. Notably, with the high transmission rates, new variants of SARS-CoV-2 have emerged, causing a new challenge in controlling this ongoing pandemic [4,5]. SARS-CoV-2 is a novel CoV that is classified within the subgenus Sarbecovirus of the genus Betacoronavirus genera, along with SARS-CoV-1 and a number of emerging animal and bat CoVs [1]. While the search for the origin of SARS-CoV-2 is still ongoing, it is believed that this virus originated from bats and was transmitted to an intermediate host before it spilled over into humans, in a scenario similar to the emergence of SARS-CoV-1 and MERS-CoV [6,7]. Like other CoVs, the genome of SARS-CoV-2 is approximately 30 kb in length and contains at least six open reading frames (ORFs) that encode for four main structural proteins (envelope (E), membrane (M), spike (S), and nucleocapsid (N) proteins) and up to 16 non-structural proteins (nsps), as well as other accessory proteins [8]. The S protein plays a critical role in attachment and entry of the virus to host cells because it contains the receptor-binding domain (RBD) that mediates virus binding to cellular receptors, and S2 which mediates fusion with cell membranes [9]. Because of this, the S protein is considered the major target for vaccine development against SARS-CoV-2 [10]. That is, almost all currently approved vaccines, including the Pfizer-BioNTech (BNT162b2) mRNA vaccine, target the S protein as an immunogen.

Vaccination is a crucial public health intervention method to control the spread of infectious diseases, including the ongoing COVID-19 pandemic [5]. Currently, nearly 190 COVID-19 vaccines are at different stages of pre-clinical and clinical development, with a few vaccines having been recently granted an Emergency Use Authorization (EUA) and are approved by WHO in many parts of the world. Among the currently approved COVID-19 vaccines are the Oxford/AstraZeneca AZD1222 and Janssen Ad26 CoV2.S which are based on non-replicating viral vector platforms; and Pfizer-BioNTech (BNT162b2) and Moderna mRNA-1273 which are based on messenger RNA (mRNA) technology [10,11,12,13].

Currently, substantial mass vaccination campaigns are underway worldwide to combat this ongoing pandemic. Globally, almost 5.5 billion doses of COVID-19 vaccines have been administered to date, with a rate of 33.54 million doses given on a daily basis [14]. Saudi Arabia was one of the first countries to initiate COVID-19 vaccination programs following approval of COVID-19 vaccines [15]. Among all other COVID-19 vaccines, the Pfizer-BioNTech COVID-19 mRNA vaccine was the first vaccine to receive approval in Saudi Arabia. The initial authorization for this vaccine was given for some high-risk and vulnerable groups, including healthcare workers and old people with chronic diseases. Thereafter, it became widely available for the whole population, excluding children and pregnant women [15]. However, recently, this vaccine was approved in Saudi Arabia for both pregnant women and children between the ages of 12–18 years, Table 1 [15,16,17]. As of October 10, 2021, there have been 44 million administered doses of COVID-19 vaccines in Saudi Arabia [17].

The Pfizer-BioNTech vaccine (BNT162b2) is based on mRNA technology to express the SARS-CoV-2 spike (S) gene [12]. The technology of mRNA vaccines has recently been introduced for its potential use in vaccine production, and some are on ongoing trials, such as vaccines against HIV and ZIKA virus [10]. However, Pfizer-BioNTech vaccine (BNT162b2) is considered the first mRNA-based vaccine for infectious diseases approved for human use [18]. BNT162b2 uses a carrier system based on lipid nanoparticles which is stabilized by a polyethylene glycol (PEG) 2000 lipid conjugate, providing a hydrophilic layer, extending its half-life [11,19]. Data from phase 3 trials revealed that two doses of BNT162b2 have a good safety profile and showed almost 95% efficacy rate against COVID-19 in persons 16 years of age or older [20,21,22]. Similarly, a phase 3 trial showed that BNT162b2 has favorable safety with transient mild-to-moderate reactogenicity and 100% efficacy rate in 12- to 15-year-old adolescents [21]. Like the older age group, the most predominant side effects reported in 12- to 15-year-old adolescents were injection-site pain (in 79 to 86% of participants), fatigue (in 60 to 66%), and headache (in 55 to 65%) [22]. Although data from clinical trials can demonstrate the efficacy and safety profiles of new vaccines, post-marketing evaluation of these vaccines is still required to assess their efficacy and safety in real-world settings. Real-word data may also provide significant assurance to the public to accept vaccines [23]. Studies in phase 3 trials may have some limitations in evaluating vaccine efficacy and safety as they recruit a small number of participants as well as a healthier than average sample population. This may result in misidentification of less frequent adverse events [20]. The rapid development of COVID-19 vaccines due to the urgency of the pandemic and the implementation of a newly emerging technique for vaccine development (i.e., mRNA vaccine) have led to some concerns regarding potential side effects [24,25,26,27,28]. Although several reports have been released on the safety and effectiveness of vaccines against SARSCoV-2, real-world data on the safety of this novel messenger RNA (mRNA)-based COVID-19 vaccine, particularly in children, are still scarce. Further, epidemiological studies, despite their known limitations, are important to evaluate the short-term COVID-19 side effects and to reassure the public about vaccine safety and effectiveness, which should not be very different from those reported in the clinical trials. Therefore, this study aimed to investigate short-term side effects after receiving the COVID-19 Pfizer-BioNTech vaccine in children aged 12–18 years in Saudi Arabia.

## 2. Materials and Methods

### 2.1. Study Design, Population, and Setting

A retrospective, cross-sectional study was conducted using a self-administered online survey to report side effects in children aged 12–18 years-old following Pfizer-BioNTech (BNT162b2) vaccination in Saudi Arabia. The survey was designed to have a dual language (Arabic and English) questionnaire, using Google Forms, which was delivered to participants via social media between 1st August to 24th August of 2021. This study included participants who received both single and double doses of the Pfizer-BioNTech (BNT162b2) vaccine. The survey was developed after an intensive literature search that included PubMed, Medline, Google Scholar, and other databases, aiming to identify potential and common short-term side effects post-Pfizer-BioNTech (BNT162b2) vaccine. The questionnaire was designed to have multiple sections. The first section has an introductory part about the purpose of the study, contact details for the study investigators to facilitate communication between study investigators and participants as well as a consent section for agreeing to participate in the study. The second section was designed to collect general information about the study participants including gender, age, chronic diseases such as hemoglobinopathies, type 1 diabetes, asthma, and others, as well as previous infection history with SARS-CoV-2 infection. The third section was formulated to focus mainly on the COVID-19 vaccine-related data such as number of doses received, side effects experienced following COVID-19 vaccine, timing, and duration of the side effects. Participants were also allowed to report no symptoms by leaving the box unchecked. We have included a subsection for side effects where we listed the most frequent side effects reported by other studies [20,29,30,31,32], including pain at the sites of injection, fatigue, headache, fever and chills, nausea, and vomiting. Additionally, we also provided a section for reporting other unlisted side effects which might have been experienced by our study participants. Further, we also questioned the participants to report doctors’ visits after vaccination, any admissions to hospitals post-vaccination, or the use of any medications taken post-vaccination.

### 2.2. Ethical Approval

Ethical approval for conducting this study was obtained from the standing ethical approval committee at Jazan University (reference number; REC42/1/087, date 22 March 2021). Consent was obtained from all participants or their tutors prior to participation in the study. The study excluded responses that did not contain the informed consents, participants who provided incomplete responses, those outside the age group of 12–18 years old, and those who received a COVID-19 vaccine other than the Pfizer-BioNTech (BNT162b2) vaccine.

### 2.3. Sample Size and Statistical Analysis

The sample size was calculated using raosoft.com. As there were no statistics available for the number of children in this age group among the total Saudi population, we used the whole country population (around 30 million) as our population size. The sample size was calculated based on a 5% margin of error, a 50% response rate, and a 95% confidence interval, for a population of 30 million inhabitants. Consequently, a sample size of 385 responses was determined to be sufficient for this study. However, to reduce sampling bias in our method as this study was based on an online questionnaire distributed via social media, we increased the sample size to include 965 participants, which is more than 2-folds of the required size. Descriptive statistics were reported for the collected data. Further descriptive analysis using t-test and chi-square test were performed for statistical purposes for all participants, and those who are presented with side effects versus those without side effects. Then, further descriptive analysis using t-test and chi-square test were done for those who received single dose versus two doses. The data were statistically analyzed using SPSS v.23 (IBM Corp., Armonk, NY, USA) with a significance level of *p* = 0.05 value.

## 3. Results

### 3.1. Characteristics of the Study Participants

A total of 965 responses were included in our study, while 50 participants were excluded from the analysis as they provided incomplete responses (i.e., providing inconsistent or incomplete responses, missing important items such as informed consent, age, number of doses, etc.). The median age for the study participants was 16 years old with almost 48% of the participants being male. Only 29% of the study participants reported that they had been previously diagnosed with COVID-19. A total of 10% of our study participants reported that they have current health conditions such as type 1 diabetes, sickle cell anemia, and asthma, while 44% of the participants reported that they had received two doses of the vaccines. Overall, almost 60% of the study participants experienced side effects. These data are also summarized in Table 2. We further categorized our study participants into two main groups, i.e., participants who experienced side effects and those who did not experience any side effects after vaccination. The two groups were then compared against each other using several parameters including age, sex, presence of health diseases, previous infections with SARS-CoV-2, and the number of doses received (Table 3). There was a significant association between the presence of post-vaccination side effects and the number of received doses (*p* = 0.003) (Table 3). Furthermore, significantly more side effects were reported among individuals with history of previous diagnosis of SARS-CoV2 (Table 3).

### 3.2. Side Effects Following Vaccinations

**Figure 1:**
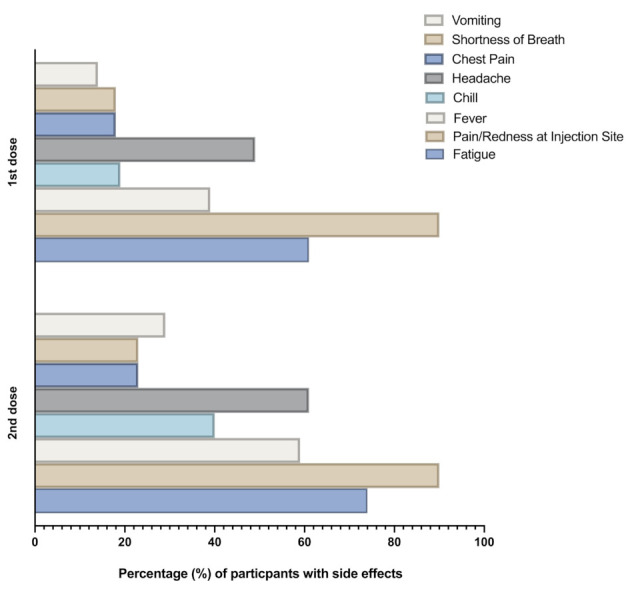
Comparison of side effects after either one or two doses of the Pfizer-BioNTech (BNT162b2) vaccine in the study participants.

In the present study, 571 of the study participants reported side effects following vaccination, representing 60% of the total study subjects (Table 2). The most frequently reported side effects among those who reported side effects after vaccination were pain or redness at the site of injection (90%), fatigue (67%), fever (59%), headache (55%), nausea or vomiting (21%), and chest pain and shortness of breath (20%). Joint or bone pain were reported less frequently among our participants (2%) (Figure 1). Almost 20% of the participants reported that the side effects started on the 2nd day following vaccination, while only 8% noticed similar side effects starting at day 3 or later post-vaccination (Table 4). These side effects lasted for 1–3 days in 65% of the participants, 3–5 days in 30% of the study subjects, and only 5% of them reported prolonged side effects, extending for more than 5 days following vaccination (Table 4). 65% of the participants reported taking painkillers to relieve the vaccine effects, with few participants needing to visit physicians or requiring hospitalization, at 14% and 8%, respectively (Table 4). Additionally, none of those who experienced side effects after vaccination reported side effects that were not listed in the questionnaire. Overall, these data indicate that more than half of the study participants who received Pfizer-BioNTech (BNT162b2) vaccine reported at least one side effect. However, these side effects were short-term in the majority of participants.

### 3.3. Comparison of Side Effects Reported Following One- or Two-Dose Vaccinations

We further categorized the participants who reported side effects following vaccination into 2 groups, i.e., participants who received single doses and participants who received two doses of Pfizer-BioNTech (BNT162b2) vaccine. These two groups were compared against each other using several parameters, including previous history of infection with SARS-CoV-2, presence of chronic health conditions, gender, time and duration of side effects, and the types of side effects experienced following vaccination (Table 5). Our data showed that side effects were commonly reported in participants who received two doses compared to the singly dosed participants. In particular, fatigue, fever, chills, headache, vomiting were the most frequently reported side effects among participants with two doses (Table 5). More participants in the two doses groups tended to take medication and were admitted to hospitals compared to singly dosed individuals, with 74% vs. 56% and 12% vs. 5%, respectively (Table 5 and Figure 1). Lastly, as shown in Table 2, reports of side effects are significantly associated with previous SARS-CoV-2 infection; here, we show that the more frequent side effects reported after the second dose compared to the first dose were neither associated with the presence of health issues nor with previous SARS-CoV-2 infection. Taken together, this data shows clearly that more significant side effects are reported after the second dose of Pfizer-BioNTech (BNT162b2) vaccine compared to the first dose in our study cohort.

## 4. Discussion

Vaccines have been proven to play critical roles in eliminating and controlling infectious diseases’ epidemics. The rapid development and availability of COVID-19 vaccines have brought a glimmer of hope to the world, and it is believed that widespread vaccination seems to be the most effective method of controlling the ongoing COVID-19 pandemic. However, vaccine acceptance and uptake could influence the efforts to control the spread of the COVID-19 pandemic. Given the rapid development and emergency authorization of COVID-19 vaccines, more trepidation about receiving COVID-19 vaccines were noticed among some populations worldwide. Furthermore, the recent reports of some rare cases of serious side effects such as thrombosis [33] and myocarditis [34,35] following COVID-19 vaccines may affect the acceptance of COVID-19 vaccines among some populations. Therefore, continuous monitoring of COVID-19 vaccines is essential to enhance the reassurance and acceptance of COVID-19 vaccines among the public. In contribution to this, we have conducted a cross-sectional, observational study to analyze the short-term adverse events following administration of Pfizer/BioNTech (BNT162b2) COVID-19 vaccines in children aged 12–18 years in Saudi Arabia.

Saudi Arabia is one of first countries that approved COVID-19 vaccines in children 12 years old and older immediately following the release of sufficient data on vaccine safety and efficacy in this age group [15,16]. In an effort to reach herd immunity as quickly as possible and to prevent SARS-CoV-2 infection in schools and universities, Saudi Arabia has made it mandatory for all persons 12 years and above to receive two doses of COVID-19 vaccines. In this web-based study, we aimed to investigate the adverse effects following administration of COVID-19 Pfizer/BioNTech (BNT162b2) vaccines in children between the age of 12–18 years old in Saudi Arabia. We found that systemic adverse effects were reported in almost 60% of our study participants. In a prior study investigating the short-term side effects of Pfizer/BioNTech in participants aged 18 to 70 years old, we have observed fewer overall report of side effects (40%, *n* = 208) [36]. More side effects have been reported by the younger population compared to their older counterparts [37,38]. This observation may be explained by the stronger and more efficient immune systems of younger people compared to older age group [19,39]. Pain/redness at the injection site (90%), fatigue (67%), fever (48%), and headache (55%) were the most frequently reported side effects by the study participants (Figure 1). The data from the present study are in support of the data from clinical trials as well as other real-world studies [20,21,22,23,24]. For example, in phase 3 clinical trials of the BNT162b2 vaccine, the three most common events after the first dose were injection-site pain (71–83%), fatigue (34–47%), and headache (25–42%) [21]. We also found that only 14% of the participants needed to see a doctor due to vaccine side effects, and only 8% of individuals were admitted to the hospital following vaccination. While the reported side effects were commonly associated with the COVID-19 vaccine, physician visits and hospitalizations after vaccination in this age group could be driven by more worrisome parents about their children.

Published literature showed that side effects were more prevalent in females than in males and in people aged 55 years or younger than in those older than 55 years [22,23,24]. Similarly, our data also showed that in children between 12–18 years, female participants reported more side effects than males. The variation in immunogenicity towards vaccinations is believed to be related to the biological differences which are imposed by gender differences [35]. Notably, a significant association between the presence of side effects and the number of received doses was also observed in the current study. More importantly, more side effects were reported in individuals with previous exposure to SARS-CoV-2 compared to those with no history of infection (Table 3). Both of these data agree with several prior reports which showed that more side effects were reported following the second doses as well as in people with a history of COVID-19 [20,21,22,23,38]. This observation could be interpreted on the basis of the immune system’s response. For instance, emerging real-world evidence indicates that the humoral immunity in individuals with prior SARS-CoV-2 infection following a single dose of the vaccine is equal to or stronger than what is found in SARS-CoV-2-naïve individuals following the second doses [40]. Such improved immunity may induce the production of cytokines that may have an inflammatory effect on muscles, blood vessels, and other tissues, mediating more systemic adverse effects post second doses or following doses in individuals who had previously been infected with SARS-CoV 2 [41]. Although it remains to be tested, this increased reactogenicity may indicate increased immunogenicity and vaccine effectiveness. However, as our work in this study is observational, we cannot directly comment on the effectiveness of the given vaccine.

To the best of our knowledge, this is the first study to investigate the adverse effects of the Pfizer-BioNTech vaccine in children aged 12–18 years old in Saudi Arabia. We have shown that the current study participants reported only common side effects which were previously reported in several other studies. Notably, we have provided our study participants with a choice to report any other side effects which were not listed in the survey. However, no other adverse effects were reported by any of the study participants, indicating that our data is in strong agreement with the type of adverse effects that were reported from phase 3 clinical study.

The data from our study showed that side effects were more frequently reported after the second doses and among individuals with a history of previous exposure to SARS-CoV-2. Results from our study may contribute to increasing the public confidence in the safety profile of COVID-19 Pfizer/BioNTech vaccines, which may contribute to accelerating the process of vaccination coverage. Our study still bears some limitations. First, the study used self-reported data, which may introduce information bias, such as inconsistency in participants’ reports, i.e., some participants might be more likely to report adverse effects than others. Additionally, the distribution of this survey was dependent on the authors’ networks. A community-based survey would be more appropriate for this kind of study. However, since social distancing is still strongly recommended, we preferred to conduct this study as a web-based study to ensure the safety of all our study participants. Another limitation of the current study is that it monitored the vaccine’s short-term side effects (immediately after injection). The medium and long-term effects of the vaccine are still unknown in our study cohort. It would be of importance for future study to assess other rare events following COVID-19 vaccination such as thromboembolic profiles and myocarditis [34].

## 5. Conclusions

In conclusion, the side effects following the COVID-19 Pfizer-BioNTech vaccine in children aged 12–18 years are common side effects which were previously reported in several other studies and do not persist for long. The adverse effects were more frequently reported among those who previously had COVID-19 and following the second doses. Data from our study may contribute to increasing public confidence regarding side effects associated with COVID-19 Pfizer/BioNTech vaccines, which may contribute to accelerating the process of vaccination coverage. A follow-up study on a larger population to evaluate the long-term side effects as well the effectiveness of the COVID-19 vaccines in children is warranted.

## Figures and Tables

**Table 1:** Approved and used COVID-19 vaccines in Saudi Arabia (data from the Saudi Public Health Authority [17]).

Manufacturer	Type	Recommended Doses	Targeted Population
Pfizer-BioNTech	mRNA	Two	Adults and children older than 12 years
Moderna	mRNA	Two	Adults and children older than 12 years
AstraZeneca-Oxford	Viral vector adenovirus	Two	18 years of age and older

**Table 2:** General characteristics of study participants.

Characteristic	Category	Number of Participants *n* (%)
Total number of participants		965
Gender	Male Female	460 (48) 505 (52)
Age ^a^		16 ± 2
Presence of health conditions	Yes No	97 (10) 868 (90)
Diagnosed previously with COVID-19	Yes	283 (29)
	No	282 (71)
Doses of Pfizer-BioNTech (BNT162b2) vaccine	One Two	539 (56) 426 (44)
Report of at least one side effect after vaccination	Yes No	571 (60) 394 (40)

^a^ Median ± SD (SD: Standard Deviation).

**Table 3:** Univariate analysis of the participants who presented with side effects compared to those without side effects following COVID-19 vaccination.

Variable	Participants with Side Effects, *n* = 571 (60%)	Participants without Side Effects, *n* = 394 (40%)	*p*-Value ^#^
Age, years (Median; SD)	16;2	16;2	1
Male, *n* (%)	274 (48)	186 (47)	0.844
Presence of health conditions, *n* (%)	56 (10)	36 (9)	0.824
Diagnosed previously with COVID-19, *n* (%)	194 (34)	89 (22)	0.0001 *
Received two doses, *n* (%)	273 (48)	153 (39)	0.003 *

SD: Standard Deviation. ^#^ The alpha criterion for *p*-value was set to 0.05. * Significant in univariate analysis.

**Table 4:** General characteristics of the participants presented with side effects after COVID-19 vaccination.

Variable	Participants with Side Effects, *n* = 571
Timing of side effects	
First day	409 (72%)
Second day	115 (20%)
Third day or later	47 (8%)
Duration of the side effects (days)	
From 1 to 3	369 (65%)
From 3 to 5	169 (30%)
More than 5	33 (5%)
Taking medication to mitigate side effects	369 (65%)
Visiting a physician due to side effects	81 (14%)
Hospitalization due to side effects	49 (8%)

SD: Standard Deviation.

**Table 5:** Comparison of side effects after one or two doses of the Pfizer-BioNTech (BNT162b2) vaccine.

Variable	Single Dose, *n* = 298 (52%)	Two Doses, *n* = 273 (48%)	*p*-Value ^#^
History of health conditions, *n* (%)	28 (9)	31 (11)	0.492
History of COVID-19, *n* (%)	102 (34)	92 (34)	0.930
Male, *n* (%)	143 (48)	131 (48)	1
Timing of side effects			0.894
First day	216	193	
Second day	60	55	
Third day or later	22	25	
Duration of the side effects (days)			0.400
From 1 to 3	200	169	
From 3 to 5	81	88	
More than 5	17	16	
Fatigue	181 (61)	201 (74)	0.001 *
Pain	269 (90)	247 (90)	1
Fever	116 (39)	161 (59)	0.0001 *
Chills	58 (19)	110 (40)	0.0001 *
Headache	146 (49)	168 (61)	0.003 *
Vomiting	42 (14)	78 (29)	0.0001 *
Chest pain	55 (18)	62 (23)	0.215
Shortness of breath	55 (18)	62 (23)	0.215
Doctor visit	35 (12)	46 (17)	0.093
Hospitalization	16 (5)	33 (12)	0.004 *
Taking medication to mitigate side effects	166 (56)	203 (74)	0.0001 *

SD: Standard Deviation. ^#^ The alpha criterion for *p*-value was set to 0.05. * Significant in univariate analysis.

## Data Availability

The dataset produced and analyzed in this study are available upon request from the corresponding author on reasonable request.
